# Arbovirus coinfection and co-transmission: A neglected public health concern?

**DOI:** 10.1371/journal.pbio.3000130

**Published:** 2019-01-22

**Authors:** Chantal B. F. Vogels, Claudia Rückert, Sean M. Cavany, T. Alex Perkins, Gregory D. Ebel, Nathan D. Grubaugh

**Affiliations:** 1 Department of Epidemiology of Microbial Diseases, Yale School of Public Health, New Haven, Connecticut, United States of America; 2 Department of Microbiology, Immunology and Pathology, College of Veterinary Medicine and Biomedical Sciences, Colorado State University, Fort Collins, Colorado, United States of America; 3 Department of Biological Sciences and Eck Institute for Global Health, University of Notre Dame, Notre Dame, Indiana, United States of America

## Abstract

Epidemiological synergy between outbreaks of viruses transmitted by *Aedes aegypti* mosquitoes, such as chikungunya, dengue, and Zika viruses, has resulted in coinfection of humans with multiple viruses. Despite the potential impact on public health, we know only little about the occurrence and consequences of such coinfections. Here, we review the impact of coinfection on clinical disease in humans, discuss the possibility for co-transmission from mosquito to human, and describe a role for modeling transmission dynamics at various levels of co-transmission. Solving the mystery of virus coinfections will reveal whether they should be viewed as a serious concern for public health.

## Background

**Fig 1:**
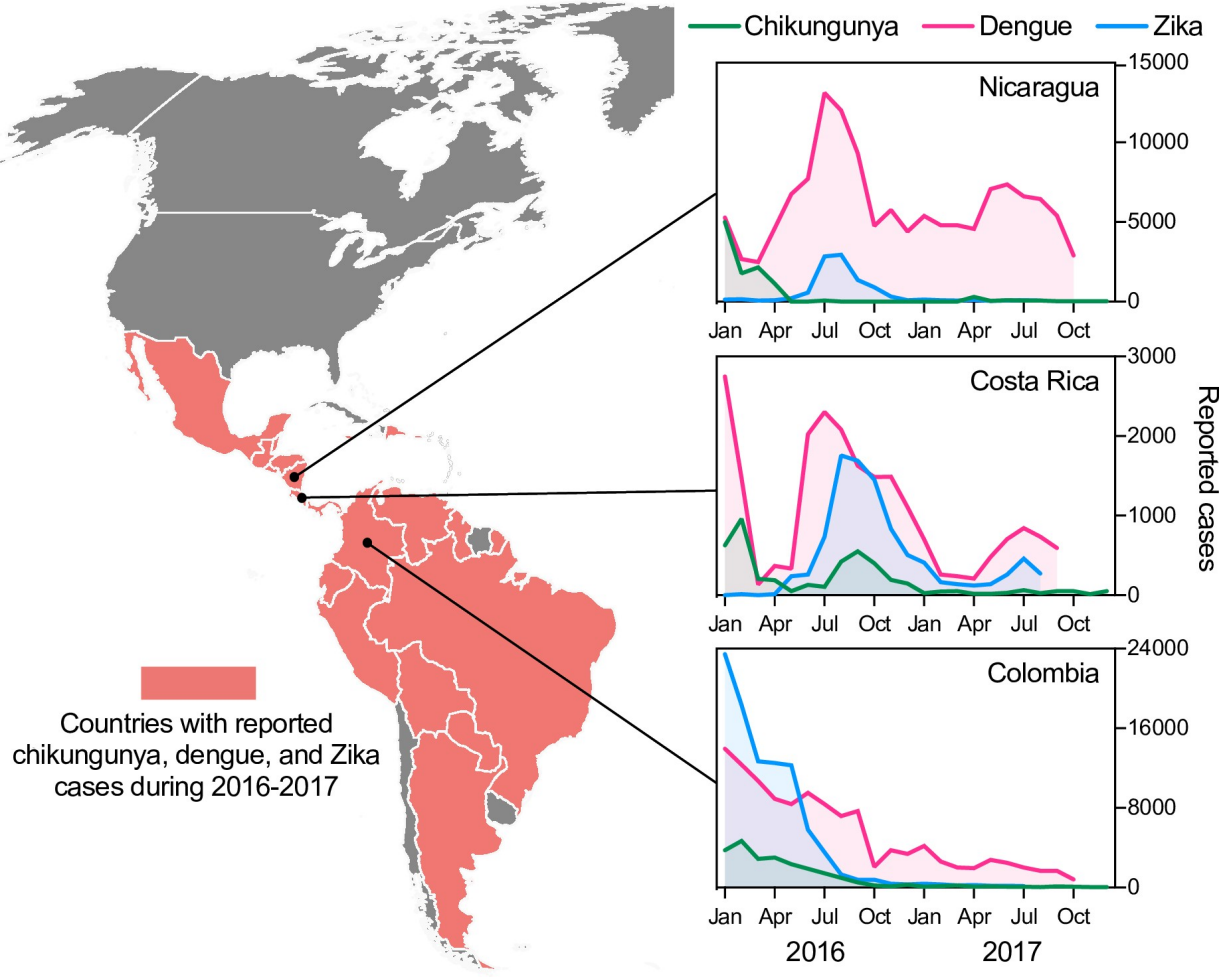
Overlapping outbreaks of chikungunya, dengue, and Zika viruses. During 2016–2017, 36 countries reported local cases of chikungunya, dengue, and Zika viruses; more, such as Cuba, Haiti, and Suriname, likely had transmission of all three viruses, but they were not reported. The inserts are examples of synergistic outbreak dynamics, shown as reported cases per month. All data were obtained from the Pan-American Health Organization and are available at https://github.com/grubaughlab/paper_2019_co-infection.

The rapid and continuous emergence of arthropod-borne viruses (arboviruses) presents a serious challenge to public health. Multiple factors, such as urbanization, increased travel, and climate change, are fueling local outbreaks and global spread [1,2]. As a result, the annual burden of dengue virus has soared to an estimated 390 million infections [3], and the recent epidemics of chikungunya and Zika viruses in the Americas may have infected hundreds of millions of people [4–6]. The emergence of chikungunya and Zika viruses in dengue-endemic regions creates intriguing, and potentially alarming, scenarios. In urban settings, all three viruses share common hosts (humans) and mosquito vectors (primarily *A*. *aegypti*) and are thus governed by similar biological, ecological, and economic factors [7], leading to epidemiological synergy [8]. So, not only do *A*. *aegypti*-borne viruses overlap geographically throughout the tropics [5,9–11], but they also have similar seasonality and attack rates. In fact, many regions in the Americas have recently experienced simultaneous outbreaks of chikungunya, dengue, and Zika virus diseases (**Fig 1**) [12,13], and concurrent infections with two or more of the viruses were commonly reported [14–25]. The surprising finding that Zika virus can cause microcephaly and other birth defects during pregnancy [26–30], the potential for dengue virus to cause severe neurological and hemorrhagic disease [13,31], and the long-term effects of chikungunya-induced chronic arthritis and cognitive disorders associated with chikungunya virus infection [5] make the potential outcomes of coinfection alarming.

As arboviruses continue to emerge, we anticipate that the occurrence of coinfection may increase as well. Despite this trend and the potential public health challenge, we know fundamentally little about the process and consequences of coinfections. Do coinfecting arboviruses alter disease in humans? Are people getting infected by multiple mosquitoes or by the same mosquito transmitting multiple viruses? Do simultaneous virus outbreaks involving the same vectors and hosts alter transmission dynamics? In this article, we use chikungunya, dengue, and Zika viruses transmitted by *A*. *aegypti* mosquitoes as a case study to describe the importance of these questions, review what data are available to help answer them, and highlight future research opportunities.

## Does coinfection alter clinical disease?

**Fig 2:**
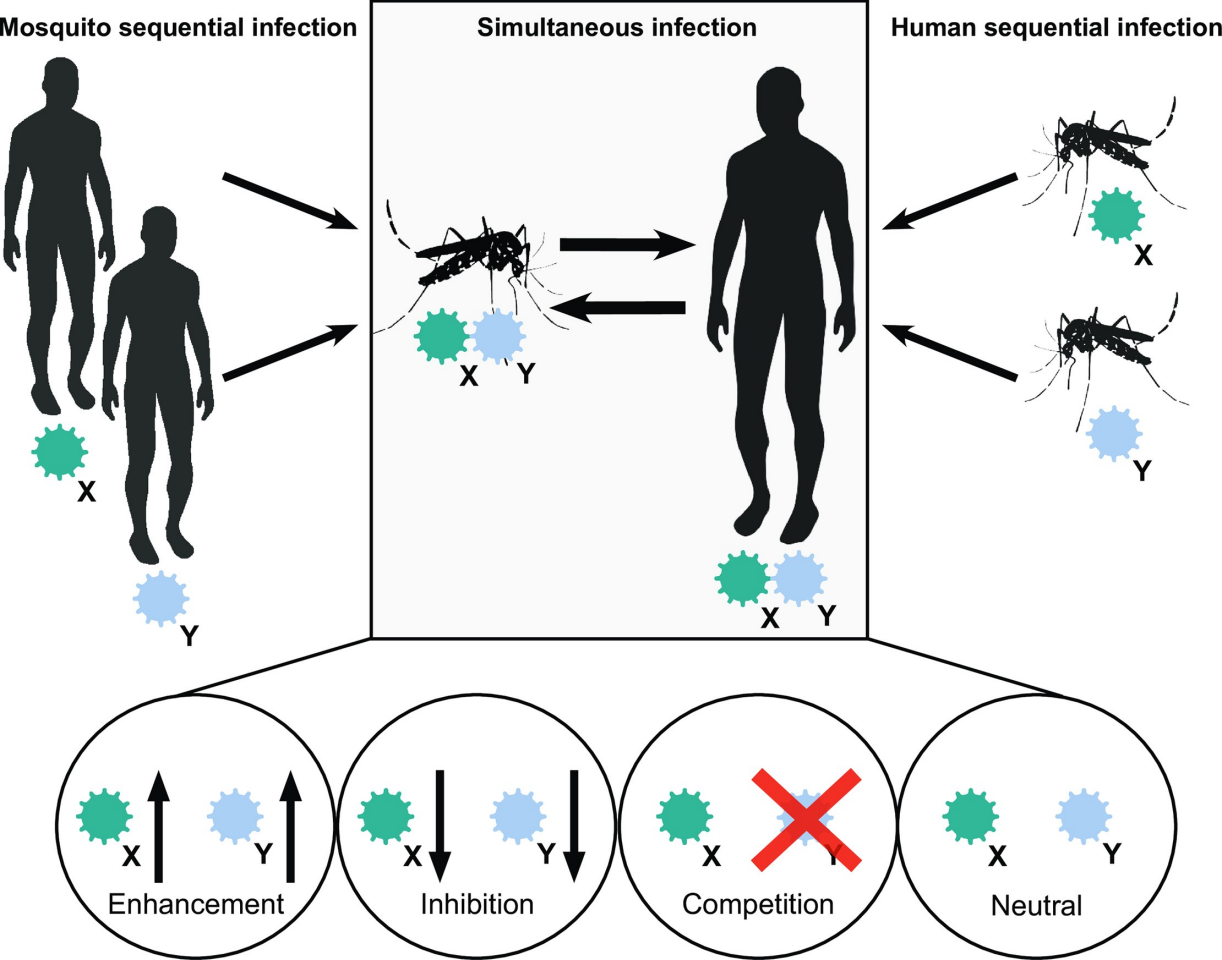
Mosquito and human coinfections occur as a result of simultaneous or sequential infection. Coinfection may either be the result of simultaneous transmission of multiple viruses between mosquitoes and humans (central panel) or sequential transmission during multiple mosquito bites. Four scenarios may explain the consequences of virus coinfection inside mosquito vectors and human hosts: enhancement, inhibition, competition, or neutral.

Assessing the public health implications of coinfection requires understanding how coinfections affect clinical disease in humans. Chikungunya, dengue, or Zika virus infections generally cause indistinguishable febrile illnesses that may include headaches, nausea, myalgia, arthralgia, and rash [13]. Some characteristic symptoms such as prolonged joint pain and swelling (chikungunya), hemorrhagic fever (dengue), and conjunctivitis (Zika) may indicate which arbovirus is causing disease, but laboratory tests are crucial for an accurate diagnosis because even these symptoms may overlap [13]. One of the major unsolved mysteries regarding coinfections is whether infection with two or more viruses can enhance disease severity compared to single infections. While the interactions of multiple infecting viruses are likely variable and complex, we anticipate four potential outcomes of coinfection: 1) enhancement of both viruses, 2) inhibition of both viruses, 3) competition between the viruses, and 4) no effect on either virus (**Fig 2**).

Enhancement of disease severity may occur if multiple arboviruses synergize and augment each other’s replication in vivo. Chikungunya, dengue, and Zika viruses infect some of the same cells, cause similar disease symptoms, and interfere with immune responses via similar mechanisms [32–34]. For instance, signal transducer and activator of transcription 1 (STAT1) and STAT2 are two transcription factors involved in interferon signaling, an important antiviral response. Thus, if chikungunya virus interferes with STAT1 nuclear transport and dengue virus blocks STAT2 phosphorylation [34], inhibiting both STAT1 and STAT2 during chikungunya/dengue coinfection may enhance replication of both viruses. As other examples, virus inhibition of 5′-3′ exoribonuclease 1 (XRN1), a cellular exonuclease that degrades viral RNA, may promote replication of a flavivirus (e.g., dengue and Zika virus) coinfecting the same cell [35], and increased endothelial permeability during dengue virus infection may alter tissue tropism of coinfecting viruses [36]. Even if coinfecting viruses do not enhance each other’s replication, coinfection may still result in increased disease severity due to exacerbated immune responses. There are some reports of severe disease after coinfection, but these are currently based only on individual case reports [37–39] or small-scale cohort studies [20,40–42]—there are no controlled animal experiments or larger cohort studies providing evidence for enhanced disease severity. Acevedo and colleagues [20], for instance, reported three patients with Guillain-Barre syndrome who were all coinfected with multiple arboviruses, including Zika virus. While these findings are concerning, none of the studies are powered to detect even fairly substantial changes in clinical presentation.

Another potential outcome is that infection with multiple arboviruses triggers a robust nonpathogenic antiviral state that reduces overall viremia and disease severity. However, identifying clinical cases providing evidence for this innocuous scenario may not be possible because of its very nature—if disease severity is significantly reduced, patients are unlikely to seek medical treatment and diagnosis for a mild fever or asymptomatic infection.

The third outcome we anticipate is competition between multiple infecting arboviruses, resulting in identical clinical presentation and transmission potential compared to single infection with the “winning” virus. Arboviruses often replicate in the same cell types (e.g., monocytes [43–45]). It is thus plausible that if one virus replicates faster upon initial infection, it can infect cells first and use up cellular resources for its own replication. Sardi and colleagues showed that the clinical symptoms mimicked infection with the virus that had a higher serum titer [17]. In addition, Zaidi and colleagues identified two cases of infants who were PCR positive for chikungunya and dengue virus at initial presentation but only seroconverted to chikungunya virus [46]. This could suggest that chikungunya virus outcompeted dengue virus early on during infection, although this effect may be confounded by the age of the patients.

**Fig 3:**
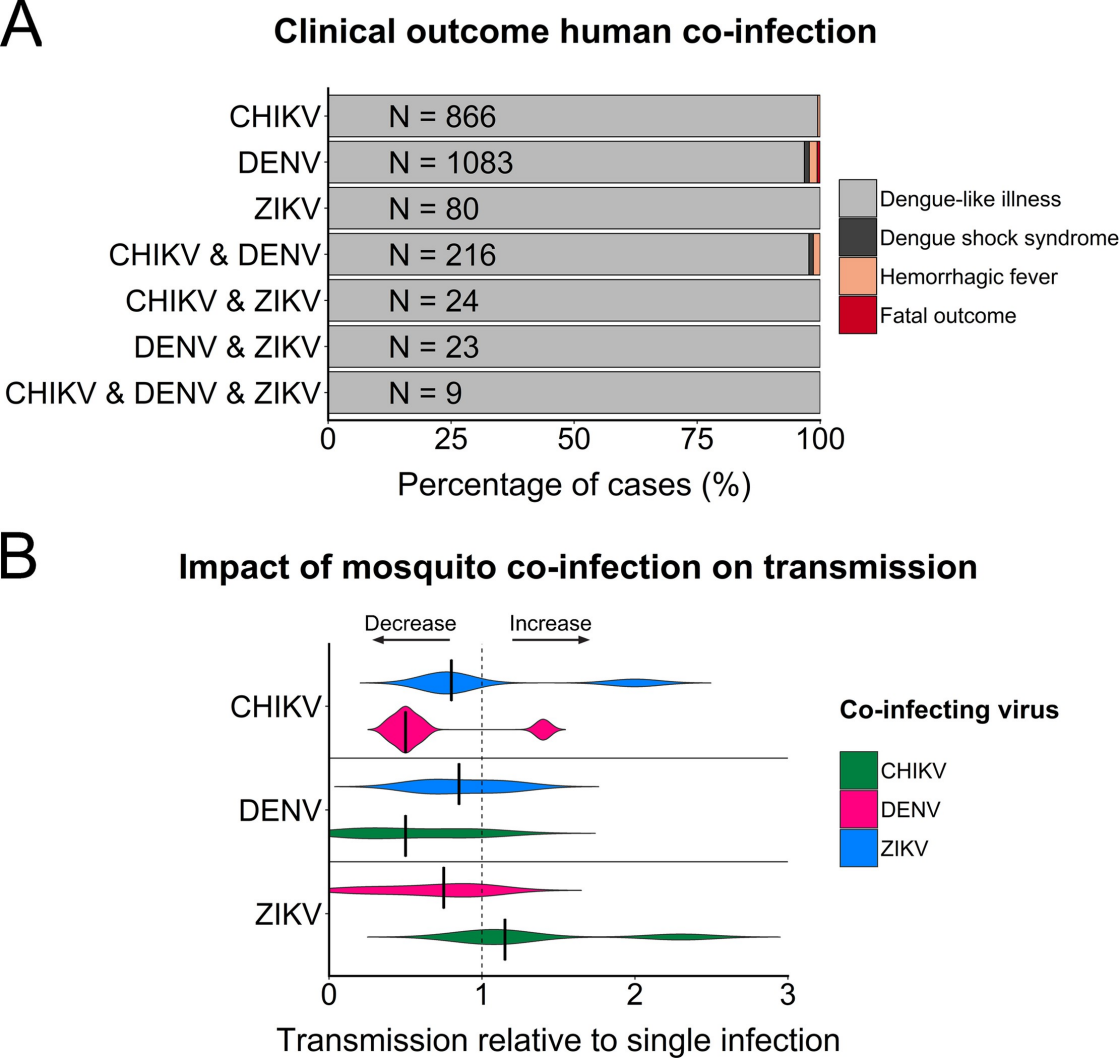
Effects of coinfection on clinical disease in humans and virus transmission by mosquitoes. (**A**) Clinical outcomes were obtained from studies providing sufficient information for both single infected and coinfected patients [22,25,32,46,47,50,52,56,58,61,63,65,67–69]. “Dengue-like illness” summarizes all cases of febrile illness with a range of additional symptoms including arthralgia, myalgia, rash, headache, gastrointestinal symptoms, thrombocytopenia, and conjunctivitis. Hemorrhagic fever includes all patients with clear signs of hemorrhage ranging from mild to severe, and dengue shock syndrome includes patients with hypotension, ascites, and pleural effusion. (**B**) Data on mosquito transmission were compiled from studies that made a direct comparison between mosquitoes exposed to a single or multiple viruses [82–84,87]. Transmission of coexposed mosquitoes was calculated relative to single exposed mosquitoes, with relative transmission being defined as transmission rate of virus X in mosquitoes coexposed to virus X and Y divided by transmission rate of virus X in single exposed mosquitoes. Transmission is expressed as the percentage of mosquitoes with virus in their saliva out of the total number of exposed mosquitoes. Relative transmission of 1 indicates that no difference was observed between transmission rates of single exposed or coexposed mosquitoes. Vertical black bars indicate the median. Data used to calculate relative co-transmission are available at https://github.com/grubaughlab/paper_2019_co-infection. CHIKV, chikungunya virus; DENV, dengue virus; ZIKV, Zika virus.

All previous scenarios imply that there is a consequential interaction between multiple arboviruses infecting the same person. It is possible, however, that coinfecting viruses can replicate within the host as if no other virus was present and that there is no significant impact on virus replication or clinical presentation—i.e., the “neutral” hypothesis. We compiled data from reports of human coinfections over the last 50 years and the clinical presentation associated with them (**S1 Table**) [14–25,32,37–42,46–72]. While severe disease manifestations occurred rarely, the majority of cases were comparable to single infections with a febrile illness, arthralgia, myalgia, and rash, most of which cleared within a week or two. Some of the more severe disease manifestations also occur in patients with single infections, such as hemorrhagic fever, encephalitis, or persistent joint pain. To directly compare single and coinfections, we compiled data from those publications that provided sufficient clinical information for both (**Fig 3A**). While these data include only a relatively small number of coinfections, disease severity was comparable between single and coinfections, with the majority of cases presenting with a dengue-like febrile illness. This could imply that coinfections either 1) have no virological or clinical impact or 2) that one virus outcompeted the other, resulting in symptoms consistent with single infection.

Despite numerous reports of arbovirus coinfections over the last few years, we are still far from knowing exactly how arbovirus coinfection impacts clinical disease—it most likely depends on the exact virus combinations, whether patients were infected simultaneously by the same mosquito or sequentially by multiple mosquitoes, and whether pre-existing antibodies or comorbidities are involved. There is some evidence that immune responses may be altered during coinfection [32,73], yet we do not know the impact this may have on disease progression. Additionally, the role of cross-reactive antibodies from previous flavivirus infection remains somewhat of a mystery both for single and coinfection [74–76]. Overall, current evidence suggests that while severe disease manifestations may occur during coinfection, these are probably not more common than severe clinical cases of single infected patients. The number of reported clinical coinfection cases remains rather low, however, and we may be missing an overall trend in altered disease progression. It is also important to note that the four scenarios we presented are not mutually exclusive, and outcomes may vary on a case-by-case basis.

## Can mosquitoes transmit multiple viruses at the same time?

Given the possible outcomes of coinfections on clinical disease, we also need to understand how humans become coinfected with multiple arboviruses. There are two possible mechanisms: 1) from simultaneous transmission of multiple viruses during a single mosquito bite or 2) from multiple sequential mosquito bites (**Fig 2**). Part of this answer lies within another intriguing question: Can mosquitoes become infected by and simultaneously transmit multiple pathogenic arboviruses?

Upon ingestion, arboviruses face multiple anatomical barriers inside the mosquito (i.e., the midgut and salivary glands) that need to be overcome before being transmitted to a new host via excretion of virus-containing saliva [77,78]. In addition to these challenges, an arbovirus must also navigate the rich microbial communities and host responses induced by these microbes inside the mosquito, which can alter transmission potential (reviewed by [79,80]). What is not entirely clear, however, are the interactions between multiple human-pathogenic arboviruses within the same mosquito vector (**Fig 2**).

By using artificial membrane feeding systems, researchers have been able to expose mosquitoes to blood meals containing one or multiple arboviruses. After keeping engorged female mosquitoes for usually 1–2 weeks at a specific temperature, saliva is collected and tested for presence of arboviruses. Detection of virus in the saliva provides a proxy for potential transmission to a new host. Such laboratory studies confirmed that mosquitoes can become simultaneously or sequentially infected with multiple arboviruses and that all combinations of chikungunya, dengue, and Zika viruses can indeed be detected in the saliva of up to 11.5% of coexposed *A*. *aegypti* mosquitoes [81–87]. Additionally, *A*. *albopictus* mosquitoes are able to co-transmit chikungunya and dengue viruses after simultaneous or sequential infection [81,82]. Thus, *A*. *aegypti* and *A*. *albopictus* mosquitoes are indeed capable of transmitting multiple arboviruses during one bite.

The four potential outcomes of coinfection that we identified for humans may also apply to mosquitoes (**Fig 2**). By comparing transmission rates of mosquitoes exposed to single or multiple arboviruses, we can identify the impact of coinfection on virus transmission. From literature, we calculated relative transmission for *A*. *aegypti* mosquitoes that were simultaneously exposed to multiple viruses, compared to single exposure [82–84,87]. Overall, relative transmission was variable, but differences between transmission rates of single infected and coinfected mosquitoes were generally small (**Fig 3)**. Thus, simultaneous coinfection of viruses in *A*. *aegypti* mosquitoes occurs without strong interference or enhancement between viruses.

Importantly, these laboratory studies were performed by simultaneously coinfecting mosquitoes, whereas in the field, mosquitoes may also become coinfected via sequential bites on different hosts. Thus far, few studies have investigated the impact of sequential coinfection on virus transmission potential [81,82,86,88]. The only enhancement that has been observed was when prior chikungunya virus infection enhanced subsequent Zika virus transmission potential in *A*. *aegypti* [88]. Additionally, only one study made a direct comparison between single infected and both simultaneously or sequentially coinfected mosquitoes with chikungunya and dengue viruses [82]. Interestingly, transmission rates of single infected and sequentially coinfected mosquitoes were comparable, whereas no evidence was found for co-transmission by mosquitoes that were simultaneously infected with chikungunya and dengue viruses. One other study also could not find evidence for coinfection after simultaneous exposure to chikungunya and dengue virus [89]. The variability in outcomes of studies may point to more complex interactions among multiple viruses, their mosquito vectors, and the environment (i.e., Genotype × Genotype × Environment interactions), which determine their transmission potential. In addition, laboratory studies may not fully reflect complexity of natural transmission dynamics, such as the secretion of nonstructural protein 1 (NS1) protein during in vivo flavivirus infection that can increase infection in mosquitoes [90].

Taken together, based on evidence from laboratory studies, we can conclude that *A*. *aegypti* and *A*. *albopictus* mosquitoes may become simultaneously or sequentially coinfected in the field and that they are able to transmit multiple viruses during one bite. Currently available data on *A*. *aegypti* suggest that simultaneous coinfection does not seem to have profound effects on transmission of individual viruses and, thus, there does not seem to be strong interference between viruses inside the mosquito or enhancement of one virus by another. However, sequential coinfection and additional mosquito–virus systems require further investigation.

## What are the epidemiological impacts of co-transmission?

The possibility for simultaneous co-transmission of multiple arboviruses between *A*. *aegypti* mosquitoes and human hosts presents another unsolved question: Can coinfections resulting from single transmission events contribute to a significantly higher burden of coinfection? The exact role that co-transmission plays in the epidemiology of coinfection will depend on arbovirus transmission dynamics between *A*. *aegypti* mosquitoes and humans, whether cross-protective immunity between viruses reduces the probability of sequential transmission, and the extent to which epidemics of different pathogens coincide in space and time.

Previous results suggest that greater than 25% of transmission events from a coinfected host or vector could result in co-transmission [84]. What implication this, or any other finding from a laboratory experiment, has for the epidemiology of arbovirus coinfections may often not be intuitively clear. To begin to assess these implications, we developed a mathematical model with basic features for relating individual-level findings about co-transmission to population-level patterns about coinfection (**Box 1**, **S1 File**). For instance, at 25% co-transmission as mentioned above, our model indicates that a majority of coinfections would result from sequential infection rather than co-transmission. If the proportion of co-transmission was higher than 42%, however, our model suggests that a majority of coinfections could be due to co-transmission, and the prevalence of coinfection would be more than double what would be expected in the absence of co-transmission (**Box 1**).

**Box 1**

We used a deterministic SIR-SI model (see **S1 File** for model details) to explore possible impacts that co-transmission from mosquitoes to humans may have on the overall dynamics of simultaneous arbovirus outbreaks. This model incorporates two viruses that have identical transmission parameters and recovery rates (for humans). The probability that transmission occurs from a coinfected human or vector is the same as if they had a single infection (i.e., coinfecting arboviruses do not affect transmission potential) [83,84]. We further assume that 60% of mosquitoes infected following a blood meal on a coinfected human become coinfected, 20% become single infected with virus X, and 20% become single infected with virus Y. In all simulations, the second virus invades one month after the first into a population of 1 million with no prior immunity. We explore three scenarios: intermediate co-transmission (50% of infectious bites from a coinfected mosquito lead to coinfection and 50% lead to single infection), no co-transmission (0% of infectious bites from a coinfected mosquito lead to coinfection and 100% lead to single infection), and all co-transmission (100% of infectious bites from a coinfected mosquito lead to coinfection and none lead to single infection).

**Fig 4:**
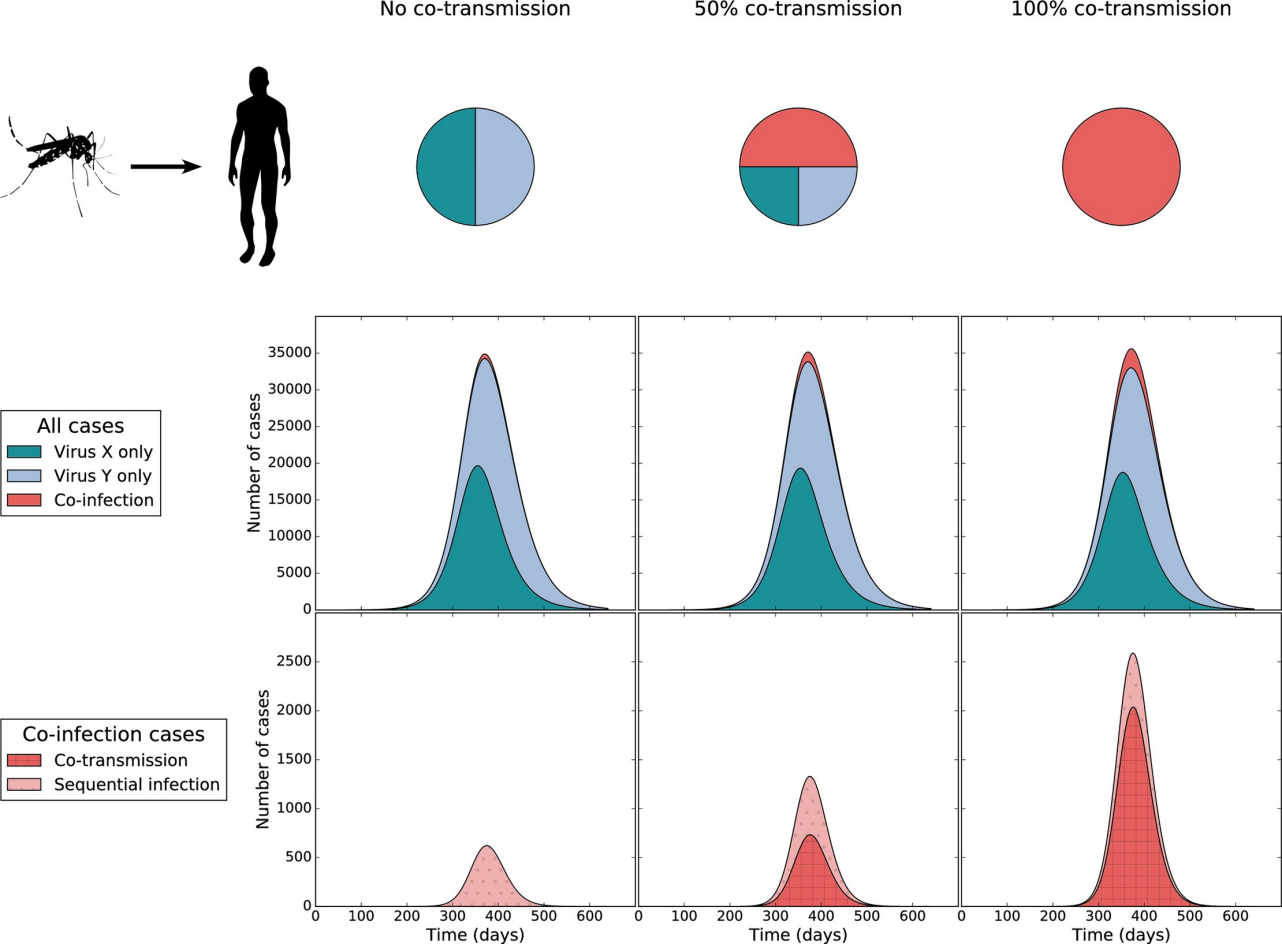
Model-predicted prevalence over time of two sequentially invading arboviruses. The transmission parameters of the viruses are identical, and virus Y invades one month after virus X in a population of 1,000,000. The top row shows the overall prevalence of both viruses and coinfection, and the bottom row shows coinfections only, delineated by the cause of the coinfection. The columns represent different scenarios of transmission from coinfected mosquitoes: no co-transmission on the left, 50% co-transmission in the middle, and 100% co-transmission on the right.

In the intermediate scenario, co-transmission has a small impact on the overall dynamics of a staggered arbovirus invasion compared to the no co-transmission scenario, with a slight increase (0.76%) in the total prevalence at the peak of the epidemic (**Fig 4**). However, there is a large increase in the burden of human coinfection, with the peak prevalence of coinfection increasing from 0.062% (1.8% of those with at least one infection) to 0.13% (3.8% of those with at least one infection), a 110% increase. Overall, the total number of human coinfections increases from 12,000 to 25,000. In the all co-transmission scenario, an unrealistic but illustrative case, we would expect an even greater increase in the burden of coinfection (**Fig 4**). In the intermediate case, 55% of all coinfections arise because of co-transmission and 45% arise because of sequential infections. In the all co-transmission scenario, 78% of coinfections arise because of co-transmission and 22% arise because of sequential infections (**S1 File**). When the probability of co-transmission, given an infectious bite by a coinfected mosquito, is 42%, we would expect half of all coinfections to be caused by co-transmission and half to be caused by sequential infections (**S1 File**). The code for this model is available at https://github.com/grubaughlab/paper_2019_co-infection.

While there is a dearth of epidemiological data on coinfections because estimating the role of co-transmission epidemiologically is difficult, some studies have attempted to estimate the proportion of cases with coinfection of those with at least one infection [9,25,46,66]. These studies vary in their design—from outbreak reports to prospective studies—and on their definition of the denominator (e.g., some use the number with dengue-like illness and others use the number with a positive test for either virus). Coinfection with chikungunya and dengue virus has been the most frequently studied. For this combination, the proportion with both viruses has been reported to be as high as 38% [46], and is often 5%–10% [9]. These high prevalences suggest that co-transmission may contribute to the observed prevalence of coinfection. Because laboratory studies have shown that mosquitoes can be simultaneously coinfected and can co-transmit, the rate of coinfections in humans raises the possibility of chains of co-transmission. Conversely, just one coinfected mosquito has ever been found in the wild, in this case coinfected with chikungunya and dengue virus [48]. While this could reflect a lack of coinfections in mosquitoes, it could also be due to infrequent mosquito surveys during simultaneous outbreaks, the fact that mosquitoes are often tested in pools rather than individually, and the low detection of mosquito infections in the field (sometimes only approximately 1:1,000 tested mosquitoes [91,92]).

A number of studies have suggested that related arboviruses display a level of cross-protection, whereby prior exposure to one virus generates an acquired response upon exposure to the second virus, which may thereby decrease the probability of sequential infections [93,94]. In the case of co-transmission to a naive host, the host will not have pre-existing immune response for either arbovirus, and cross-reactivity may play a reduced role, implying co-transmission would play a greater role in the dynamics of coinfection. This effect would likely increase the role of co-transmission in generating coinfections and may also increase the total number of coinfections (**Box 1**).

A related issue is whether coinfecting viruses influence the transmission fitness of the other (**Fig 2**). Here, there is again conflicting evidence. Zaidi and colleagues saw a significant reduction in dengue virus production and a significant increase in chikungunya production in coinfected cells, whereas Silva and colleagues saw the opposite: a significant reduction in chikungunya production but a moderate increase in dengue virus production [46,73]. Waggoner and colleagues found that during coinfections, Zika virus typically has lower viremia than either of the other two, and coinfection typically has lower viremia of either virus than single infection [25]. If one virus has higher transmission fitness than the other, this would lead to more single infections and fewer coinfections because coinfected mosquitoes/humans disproportionately transmit the more fit virus. If the transmission fitness of both viruses remains the same or is mutually enhanced, then we might expect a greater proportion of co-transmissions.

Our model simulations raise the possibility that co-transmission from *A*. *aegypti* mosquitoes to humans may make an important contribution to the burden of coinfection (e.g., a majority of the cases in the 50% co-transmission scenario) during overlapping outbreaks. The size of this contribution will be determined in part by the effect that coinfection has on the transmission potential of each virus and on the probability of co-transmission. At this point, this effect is still unclear. The number of unknowns surrounding the clinical consequences of coinfection, and whether we are accurately diagnosing coinfections (e.g., [95]), would urge us to study the potential epidemiological impacts of co-transmission carefully.

## How do we solve the mysteries of coinfection and co-transmission?

Several important questions remain to be answered. It remains unclear whether the presence of multiple infecting arboviruses within a patient impacts short- and/or long-term clinical outcomes, including for developing fetuses and in the context of highly prevalent comorbidities. Cohort studies adequately powered to detect rare events such as coinfection, including rare outcomes of these rare events, are needed in order to fully evaluate the clinical implications of coinfection and to understand the possible impact on congenital disease. Importantly, several studies have recently been initiated in endemic areas. In addition, as mouse models of arbovirus diseases improve (e.g., [96]), so may their ability to uncover the mechanisms through which these agents synergize or interfere with one another. In the coming years, the combination of large epidemiological studies and laboratory work on vertebrate immunity may shed light on what is in store as conditions worsen in the tropics and elsewhere.

Similarly, how coinfection impacts arbovirus transmission by mosquitoes is not well characterized but may have significant impacts on arbovirus transmission dynamics. It has become fairly clear that *A*. *aegypti* mosquitoes have the ability to acquire and transmit multiple arboviruses simultaneously [81–87] and that when acquired during a single feeding episode, neither viral synergism nor competition seems apparent. However, in nature, many mosquitoes may be exposed during sequential blood meals. Whether and how viruses interact in the context of sequential infection requires further study. The possibility for vertical transmission of multiple viruses also needs to be addressed, although the low rates of vertical transmission for single viruses suggest that this may contribute little in the field [97,98]. Additionally, while we focus mainly on *A*. *aegypti* and chikungunya, dengue, and Zika viruses, coinfections in other systems may also be important and require further investigation. Careful and comprehensive studies of coinfection in mosquitoes are required to fully understand how the altered pathogen landscape in the tropics may contribute to current and future virus emergence.

Finally, because of extreme environmental changes that are occurring in the tropics, the selective environment faced by *A*. *aegypti*-borne viruses appears to be fundamentally different from what existed previously. It may be that these changes, including the rise of a much more complex set of viruses that can coinfect both mosquitoes and people, will alter the rates of arbovirus evolution, as well as the evolution of virulence [99]. Experimental investigations of within-host competition and selection during virus coinfection are needed to uncover the extent to which these rare but potentially abrupt events may shape arbovirus evolutionary dynamics (i.e., the theory of punctuated equilibria).

The rise of massive urban centers in the tropics inhabited by vulnerable populations; the increased frequency and speed of intra- and intercontinental movements of humans, animals, and other materials; and the spread of human-associated mosquitoes that are resistant to many insecticides has created an unprecedented set of environments that facilitate the intense transmission of mosquito-borne infections. A detailed understanding of how coinfection impacts the biology and epidemiology of arboviruses is critical to our response to these now global pathogens because more are surely on the way.

## Supporting information

S1 Table Comprehensive list of chikungunya, dengue, and Zika virus human coinfection reports. (XLSX)

S1 File Model description and details and supplemental figures. Contains S1–S4 Figs and S2–S3 Tables.
(PDF)

## Abbreviations

CHIKV: chikungunya virus
DENV: dengue virus
NS1: nonstructural protein 1
STAT1: signal transducer and activator of transcription 1
STAT2: signal transducer and activator of transcription 2
XRN1: 5′-3′ exoribonuclease 1
ZIKV: Zika virus
