# Supplementary material for: Arbovirus coinfection and co-transmission: A neglected public health concern?
Source: PLoS Biol. 2019 Jan 22;17(1):e3000130. doi: 10.1371/journal.pbio.3000130 (PMC6358106; doi:10.1371/journal.pbio.3000130)
Supplement: S1 File — Contains S1–S4 Figs and S2–S3 Tables. (PDF) [file pbio.3000130.s002.pdf]

Model description

To explore questions related to co-transmission, we built a deterministic SIR-SI model. In this model, both humans and mosquitoes (i.e. *Aedes aegypti*) can be co-infected, and co-infected mosquitoes or humans can then transmit a single virus or multiple viruses. We model births and deaths in the mosquito population at a constant rate, thereby achieving a constant population size. Due to the timescale of interest for this illustration, we do not model human births or deaths. Cross-protective immunity is incorporated into the model as a reduction in the probability of transmission to humans who are single-infected or recovered from one virus. Humans recover from infections at a constant rate, and for simplicity, humans who are sequentially infected are assumed to be co-infected for the duration of their subsequent co-infectious period (this will lead to an overestimate of the proportion of the population in the I_{12}^S compartment). For simplicity, and because we are mainly interested in exploring the influence of variation in the probability of co-transmission, virus X and virus Y have identical parameters, except that one virus is introduced one month after the other. Other transmission parameters (see **S2 Table** for details) were chosen to be broadly in line with previous dengue modeling studies [1–3], and to produce a final attack rate of ~60% for a single invading virus.

Model equations for humans are as follows, with parameter values and their meaning given in **S2 Table**:

$$\frac{dS}{dt} = -(\lambda_1 + \lambda_2 + \lambda_{12})S$$

$$\frac{dI_1}{dt} = \lambda_1 S - (\lambda_2 + \lambda_{12} + \frac{1}{r})I_1$$

$$\frac{dI_2}{dt} = \lambda_2 S - (\lambda_1 + \lambda_{12} + \frac{1}{r})I_2$$

$$\frac{dI_{12}{}^C}{dt} = \lambda_{12}(S + I_1 + I_2) - \frac{I_{12}{}^C}{r}$$

$$\frac{dI_{12}{}^S}{dt} = \lambda_2 I_1 + \lambda_1 I_2 - \frac{I_{12}{}^S}{r}$$

$$\frac{dI_{1,2}}{dt} = (\lambda_{12} + \lambda_2)R_1 - \frac{I_{1,2}}{r}$$

$$\frac{dI_{2,1}}{dt} = (\lambda_{12} + \lambda_1)R_2 - \frac{I_{2,1}}{r}$$

$$\frac{dR_1}{dt} = \frac{I_1}{r} - (\lambda_{12} + \lambda_2)R_1$$

$$\frac{dR_2}{dt} = \frac{I_2}{r} - (\lambda_{12} + \lambda_1)R_2$$

$$\frac{dR_{12}}{dt} = \frac{1}{r}(I_{1,2} + I_{2,1} + I_{12}{}^C + I_{12}{}^S)$$

$$\lambda_1 = mab(I_1{}^M + p_1 I_{12}{}^M)$$

$$\lambda_2 = mab(I_2{}^M + p_2 I_{12}{}^M)$$

$$\lambda_{12} = mabp_{12} I_{12}{}^M$$

Equation for mosquitoes are as follows, with parameter values and their meaning given in **S2 Table**:

$$\frac{dS^M}{dt} = g(I_1{}^M + I_2{}^M + I_{12}{}^M) - (\lambda_1{}^M + \lambda_2{}^M + \lambda_{12}{}^M)S^M$$

$$\frac{dI_1{}^M}{dt} = \lambda_1{}^M S^M - (\lambda_2{}^M + \lambda_{12}{}^M)I_1{}^M - gI_1{}^M$$

$$\frac{dI_2{}^M}{dt} = \lambda_2{}^M S^M - (\lambda_1{}^M + \lambda_{12}{}^M)I_2{}^M - gI_2{}^M$$

$$\frac{dI_{12}{}^M}{dt} = \lambda_{12}{}^M S^M + (\lambda_1{}^M + \lambda_{12}{}^M)I_2{}^M + (\lambda_2{}^M + \lambda_{12}{}^M)I_1{}^M - gI_{12}{}^M$$

$$\lambda_1{}^M = ac(I_1 + I_{2,1} + p_1{}^M(I_{12}{}^C + I_{12}{}^S))$$

$$\lambda_2{}^M = ac(I_2 + I_{1,2} + p_2{}^M(I_{12}{}^C + I_{12}{}^S))$$

$$\lambda_{12}{}^M = acp_{12}{}^M(I_{12}{}^C + I_{12}{}^S)$$

S1-S2 Figs provide a graphical representation of the state transitions defined by the equations.

Simulations of the model are initiated by introducing a single infectious human into a population of 1,000,000. After 30 days, a single person with virus 2 is introduced into the population. We assume that there is no cross-protective immunity between the two viruses.

S3 Fig shows how the total number of co-infections over the course of the outbreak changes as we vary p_1, p_2, and p_{12}. The highest number of co-infections occur when p_{12} is highest (bottom left corner), which is also when p_1 and p_2 are lowest as $p_1 + p_2 + p_{12} = 1$.

S4 Fig shows how the number of co-infections (left panel) and the proportion of co-infections due to co-transmission (right panel) varies as we change the probability of co-transmission, p_{12}, and keep $p_1 = p_2$. more than half of co-infections are due to co-transmission when p_{12} =0.175 (right panel).

Probabilities of co-transmission

A rough estimate of the probabilities of co-transmission from co-infected humans to mosquitoes can be obtained with data from Rückert et al. [4]. This study fed mosquitoes on blood that contained a single virus or all combinations of chikungunya, dengue, and Zika viruses. They then tested which mosquitoes were single- or co-infected, and also which mosquitoes had either or both virus in their saliva after 3, 7, and 14 days (a proxy for transmission potential).

For mosquitoes co-exposed to dengue and Zika virus, there were 197 mosquitoes that were infected, of which 118 (60%) were co-infected. Similarly, using the data underlying Figure 4 in Rückert et al. [4], after 14 days there were 26 dengue/Zika virus co-infected mosquitoes which had at least one virus in their saliva, of which 7 (27%) had both. The equivalent figures for the other pairs of viruses are shown in **S3 Table.** For simplicity of analysis, our model assumes that virus 1 and 2 have equal probabilities of co-transmission ($p_1 = p_2$), although in practice it seems likely these values will differ depending on the pair of viruses in question (**S3 Table**).

Supplemental tables

S2 Table. Parameter names, meanings and values. See the next section of the appendix for a discussion of the probabilities of transmission from co-infected *Ae. aegypti* mosquitoes and humans. Transmission parameters specific for *Ae. aegypti* mosquitoes were chosen broadly in line with previous dengue modeling studies [1–3], and to produce a final attack rate of 60% for a single invading virus.

Parameter	Meaning	Value
m	Ratio of *Ae. aegypti* mosquitoes to humans	1.0
a	Mosquito biting rate	0.5/day
b	Probability that an infected mosquito transmits to a human during feeding	0.3
c	Probability that a mosquito becomes infected after feeding on an infected human	0.5
r	Average time for a human to recover	5 days
g	Mosquito mortality rate	0.125/day
p_1	Probability that when a co-infected mosquito transmits, it transmits only virus X to a human	varied
p_2	Probability that when a co-infected mosquito transmits, it transmits only virus Y to a human	varied
p_{12}	Probability that when a co-infected mosquito transmits, it transmits both viruses to a human	varied
$p_1{}^M$	Probability that when a mosquito becomes infected after feeding on a co-infected human, it only becomes infected by virus X	0.2
$p_2{}^M$	Probability that when a mosquito becomes infected after feeding on a co-infected human, it only becomes infected by virus Y	0.2
$p_{12}{}^M$	Probability that when a mosquito becomes infected after feeding on a co-infected human, it becomes infected by both viruses	0.6

S3 Table. Proportion of transmission events leading to co-infection from a co-infected human to a mosquito ($p_{12}{}^{M}$) or from a co-infected mosquito to a human (p_{12}). In the latter case we assume that co-infection does not affect the transmission probability, and that hence co-transmission occurs in the same proportion as which it is found in the saliva.

**Virus 1**	**Virus 2**	$p_{12}{}^{M}$	p_{12}
Dengue	Zika	0.60	0.70
Dengue	Chikungunya	0.77	0.38
Zika	Chikungunya	0.36	0.27

Supplemental figures

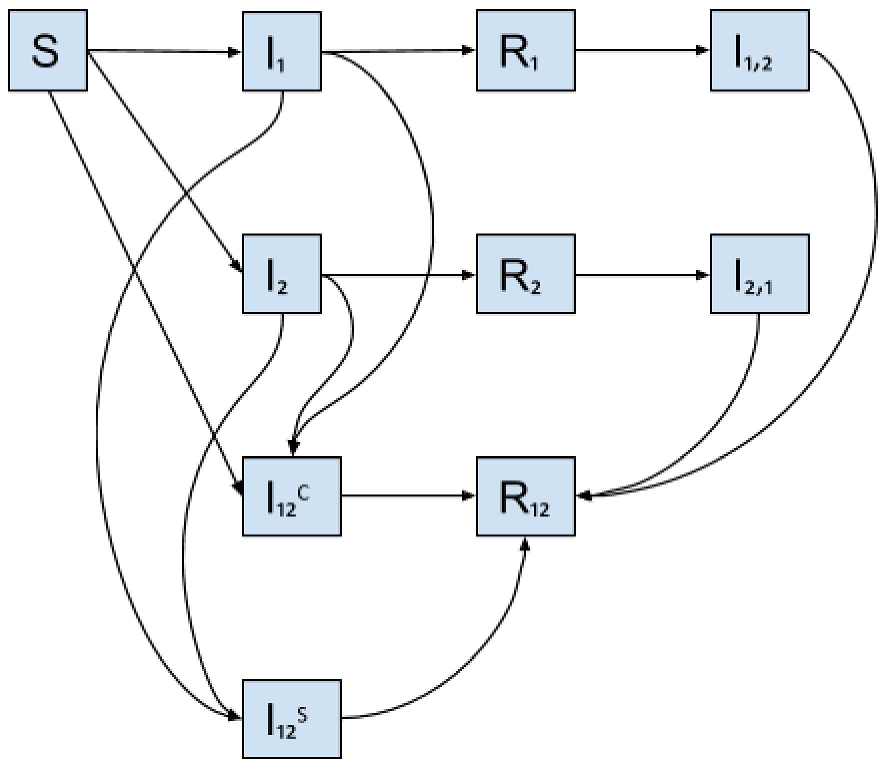

S1 Fig. Model diagram for the human component of the model. Susceptible individuals (S) can be infected by virus 1 (from either a mosquito with virus 1 or from a co-infected mosquito), by virus 2 (from either a mosquito infected with virus 2 or a co-infected mosquito), or by both viruses from a co-infected mosquito. Single-infected humans (I_1 and I_2) can either be infected by the other virus and become co-infected, or recover. Co-infected humans can be co-infected either due to co-transmission (I_{12}^C) or due to sequential transmission (I_{12}^S), and then recover. Individuals who have recovered from just one virus (R_1 and R_2) can become infected with the other virus ($I_{1,2}$ and $I_{2,1}$) and are then infectious with only their second infection. Individuals who have recovered from both infections (R_{12}) form an absorbing state and remain immune.

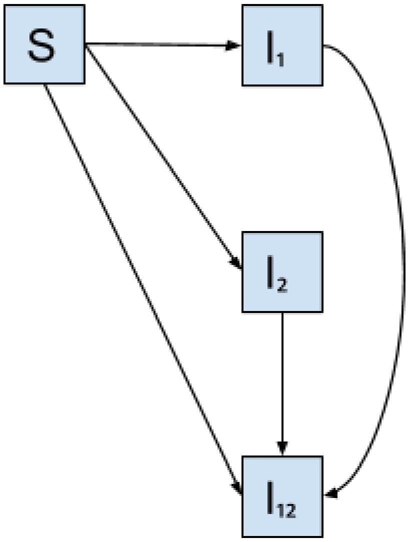

S2 Fig. Model diagram for the mosquito component of the model. Susceptible mosquitoes (S) can be infected by virus 1 (from either a human with virus 1 or from a co-infected human), by virus 2 (from either a human infected with virus 2 or a co-infected human), or by both viruses from a co-infected human. Single-infected mosquitoes (I_1 and I_2) can be infected by the other virus, becoming co-infected. Mosquitoes remain infected for the duration of their lifetime. From all states, mosquitoes die at a constant rate, and are born into the susceptible compartment at the same rate to maintain a constant population. All infected mosquitoes transmit with probability *c* per bite, and co-infected mosquitoes transmit virus X, virus Y, or both, in a proportion given by $p_1{}^M$, $p_2{}^M$, and $p_{12}{}^M$.

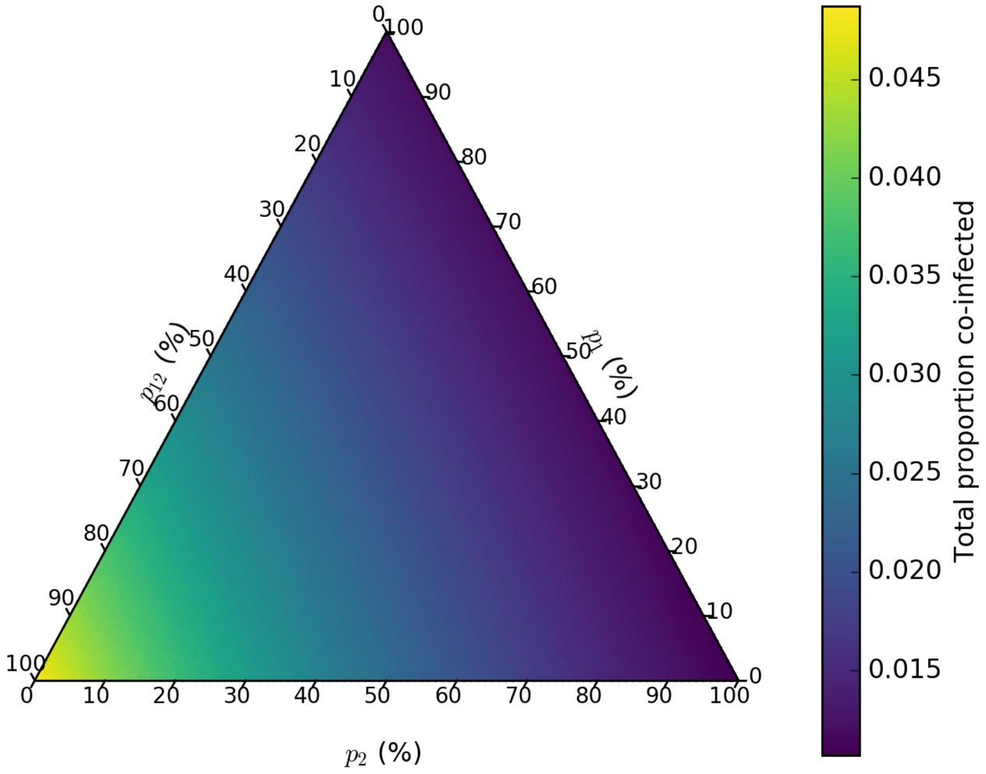

S3 Fig. Total proportion of individuals that were co-infected by the end of the epidemic. This shows the final attack rate with changing probabilities of co-transmission. p_{12} represents the probability of co-transmission given an infectious bite by a co-infected mosquito.

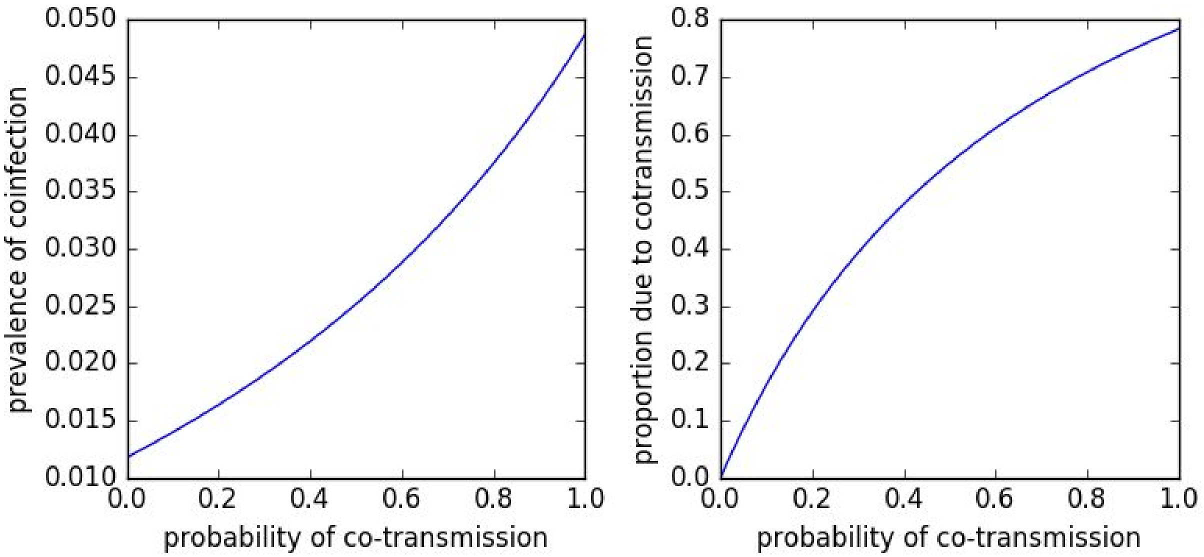

S4 Fig. The impact of varying the probability of co-transmission on both the prevalence of co-infection (left) , and the proportion of co-infection that is due to co-transmission (right) .

Supplemental references

1. Newton EAC, Reiter P. A model of the transmission of dengue fever with an evaluation of the impact of ultra-low volume (ULV) insecticide applications on dengue epidemics. Am J Trop Med Hyg. 1992;47: 709–720.

2. Burattini MN, Chen M, Chow A, Coutinho FAB, Goh KT, Lopez LF, et al. Modelling the control strategies against dengue in Singapore. Epidemiol Infect. 2007;136: 309–319.

3. Flasche S, Jit M, Rodríguez-Barraquer I, Coudeville L, Recker M, Koelle K, et al. The long-term safety, public health impact, and cost-effectiveness of routine vaccination with a recombinant, live-attenuated dengue vaccine (Dengvaxia): A model comparison study. PLoS Med. 2016;13: e1002181.

4. Rückert C, Weger-Lucarelli J, Garcia-Luna SM, Young MC, Byas AD, Murrieta RA, et al. Impact of simultaneous exposure to arboviruses on infection and transmission by *Aedes aegypti* mosquitoes. Nat Commun. 2017;8: 15412.
